# The Effect of Organic Inhibitors on the Corrosion of Mild Steel in Varying Crude Oil–Brine Emulsions: The Electrochemical Protocol Selection

**DOI:** 10.3390/molecules31203743

**Published:** 2026-10-18

**Authors:** Nataliya A. Ivanova, Adina Alibayeva, Xeniya Leontyeva, Nurislam Shaiakhmetov, Batyr Boribaiuly, Guzal Ismailova, Ruslan Nurislamov, Gulinur Khussurova, Vladislav Kudryashov

**Affiliations:** 1 D.V. Sokolsky Institute of Fuel, Catalysis and Electrochemistry JSC, 142 Kunayev Str., Almaty 050010, Kazakhstan; ivanovana.1989@outlook.com (N.A.I.); adinaalibayeva10@gmail.com (A.A.); leontyeva.xeniya@mail.ru (X.L.); nurislamshaiakhmetov@gmail.com (N.S.); bmursaitov@mail.ru (B.B.); ruslan.nur@gmail.com (R.N.); gulinur_k@bk.ru (G.K.); 2 Al-Farabi Kazakh National University, 71, Al-Farabi Avenue, Almaty 050012, Kazakhstan; guzal2097@gmail.com; 3 Kazakh British Technical University, Tole bi Street, 59, Almaty 050000, Kazakhstan; 4 Renewable Energy Lab, “National laboratory Astana”, Nazarbayev University, 53 Kabanbay Batyr Ave., Astana 010000, Kazakhstan

**Keywords:** steel corrosion, corrosion rate, organic corrosion inhibitors, oil–brine electrolyte, direct and inverse emulsions, electrochemical protocol limitations

## Abstract

Corrosion inhibitors are widely used in oil production, transportation, and refining, where crude oil–brine emulsions complicate electrochemical testing because of their heterogeneity and low conductivity. This study examined the use of Tafel polarization and electrochemical impedance spectroscopy to assess mild steel corrosion in brine and crude oil–brine emulsions in the presence of Nalco EC 1010B and Scimol OR 2001. Emulsions with a continuous conductive phase were tested directly, whereas the brine phase of reverse emulsions was separated before measurement. In brine, both inhibitors provided more than 80% protection at a concentration of 20 mg/L. At this concentration, corrosion rates in the inhibited direct emulsion and the brine phase separated from the reverse emulsion were approximately 0.20–0.25 mm/year, comparable to those in inhibited brine under similar conditions. In stable emulsions, the surfactant also affected steel corrosion, complicating the assessment of inhibitor performance. These results indicate that measurements in brine can guide the selection of inhibitor concentrations for direct and reverse emulsions under the conditions investigated.

## 1. Introduction

In the modern petroleum refining industry, corrosion remains a major cause of technical and economic losses, accounting for up to 10% of GDP annually [1,2,3,4,5]. Corrosion and erosion-corrosion pose challenges to the integrity of metallic equipment used in oil production, transportation, and refining [6,7,8,9]. Complex, multistage processes give rise to a range of conditions with varying corrosion activity, including those in pipelines, which form the backbone of fluid transport infrastructure in the energy sector [1,2,10]. The multicomponent nature of these systems requires investigation of how each constituent of the corrosive medium affects its overall corrosivity, as well as the identification of conditions associated with increased corrosion of structural metals. The combined presence of oxygen, water, and salts, including Cl^−^ and HCO_3_^−^ ions, together with elevated concentrations of sulfur-containing compounds and hydrocarbon impurities [8,10,11,12,13], leads to the corrosion of structural steels. The relationship between the steel corrosion rate and the properties of the transported fluid is complex and non-linear [7].

Depending on the ratio of the aqueous phase, including brine, to petroleum fractions, three types of emulsions with different compositions and properties can be distinguished: direct emulsions (oil-in-water), reverse emulsions (water-in-oil), and stable emulsions (micellar solutions). Direct and reverse emulsions are most commonly encountered in oil production and crude oil transportation [14]. Stable emulsions, however, are more suitable for electrochemical studies and corrosion testing. Previous studies have reported that the formation of an oil-phase layer composed of light hydrocarbon fractions on the steel surface can reduce corrosion rates. Conversely, corrosion rates increase significantly when oil–water emulsions become destabilized [15,16,17,18]. The available data remain fragmented and lack systematic consistency, indicating a need for further investigation and interpretation. This need is particularly relevant to oil–water emulsions containing crude oil fractions, as crude oil systems have been shown to exert a more pronounced influence on corrosion processes than refined petroleum products [3]. Understanding the corrosion behavior of steels in oil-in-water emulsions is important for identifying the underlying corrosion mechanisms [1,6,14,19,20,21,22] and developing effective corrosion inhibitors for pipeline protection [15,16,17]. The design of such inhibitor systems requires consideration of the characteristics of the corrosive medium, including whether it is acidic, neutral, or alkaline, its ionic composition, and the nature of the inhibitor [10,23,24], as well as potential interactions between the inhibitor formulation and constituents of the oil phase [7,25,26].

Electrochemical testing is an established method for rapidly assessing the corrosion behavior of steels in various environments, including brine, and evaluating inhibition efficiency. Tafel-plot analysis (a Tafel-based potentiostatic approach) and electrochemical impedance spectroscopy [5,18,27,28,29,30] provide information on corrosion kinetics and the performance of organic inhibitors [6,13,14,17,19,26,31]. Their application to steel alloys in emulsions of heavy petroleum fractions and salt solutions is complicated by the heterogeneity and low electrical conductivity of the electrolyte [5,13,18,28,29]. Additional components are often introduced to improve emulsion stability and electrical transport. Such modifications, however, alter the corrosion characteristics of the system and introduce uncertainty when inhibitor performance is extrapolated to real, non-stabilized emulsions [12].

The electrochemical and corrosion behavior of steels in emulsified systems is therefore important for understanding how the properties of oil–brine electrolytes influence corrosion rates and mechanisms. This understanding is also needed to evaluate corrosion-inhibiting formulations intended to extend the service life of petroleum transport systems. The present study aims to identify the limitations of electrochemical methods for determining steel corrosion rates in complex emulsified media and to adapt electrochemical protocols for studying corrosion in the presence of organic inhibitors without altering the composition or corrosivity of the media. Our hypothesis is that testing organic steel corrosion inhibitors in stable brine–oil emulsions may produce erroneous data because of the surfactants present in the multicomponent electrolyte. The selection of suitable electrolyte pretreatment methods facilitates the evaluation of steel corrosion protection in both direct and reverse brine–oil emulsions.

## 2. Results

### 2.1. The Effective Concentration of Nalco EC 1010B and Scimol OR 2001 Inhibitors in Brine

**Figure 1:**
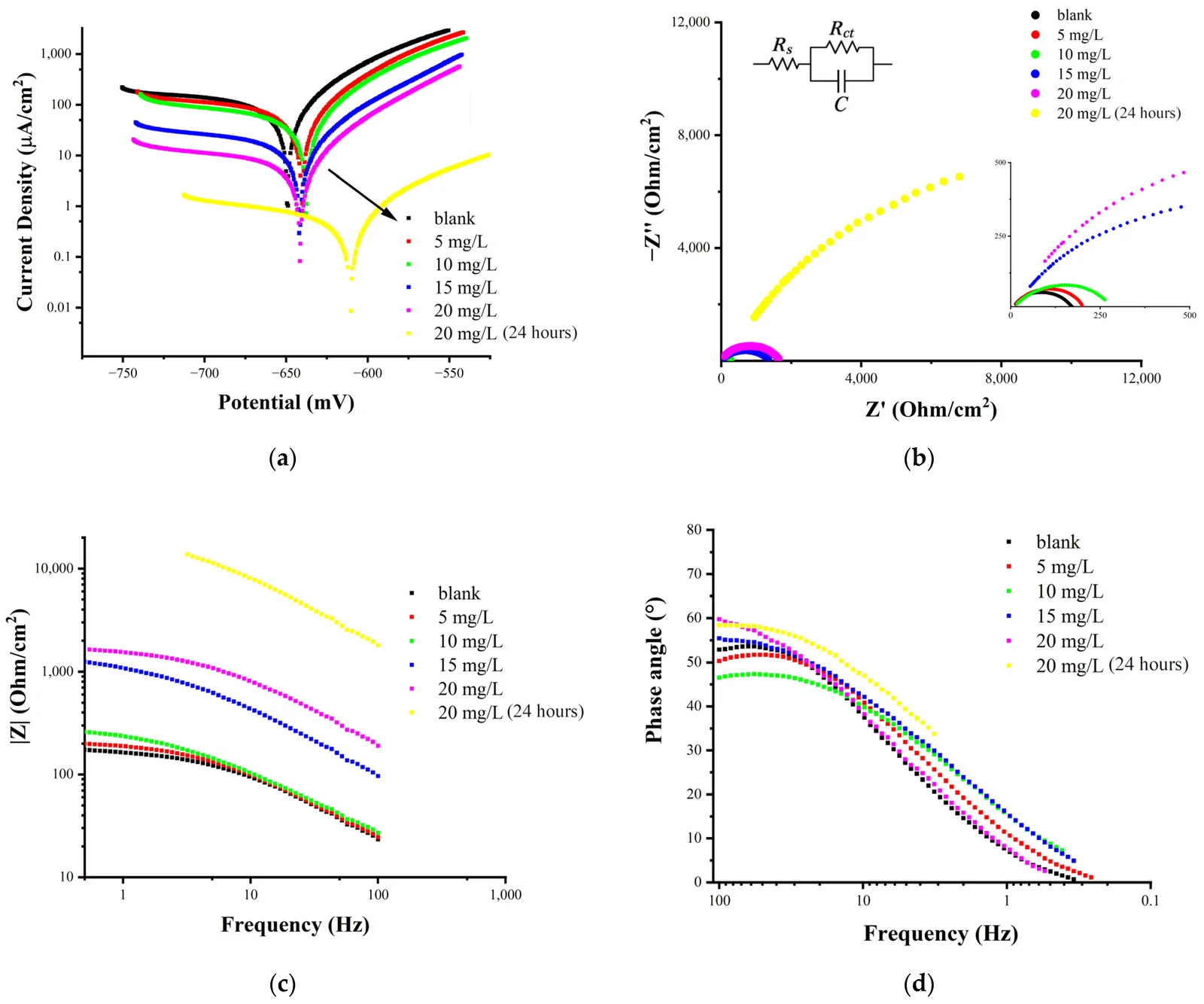
Tafel (**a**), Nyquist (**b**), Bode (**c**) and Phase angle (**d**) plots of steel coupons in brine in the presence of the Nalco EC 1010B inhibitor.

Figure 1 presents the Tafel (a), Nyquist (b), Bode (c), and phase-angle (d) plots for mild steel coupons in brine containing the Nalco EC 1010B inhibitor. Because the main inhibitory effect of the mixture arises from the slow physical adsorption of the reagents onto the steel surface, the formation of a strong, continuous film takes longer than chemical film formation involving complexes of the substance or corrosion products [10,31]. Electrochemical parameters were therefore recorded immediately after cell assembly and after the test medium had been maintained with the inhibitor for 24 h without stirring. The electrochemical measurements and corrosion rates calculated from the Tafel plots are presented in Table A1, and the EIS results are presented in Table A2.

The steel corrosion rate decreases gradually from 1.8 ± 0.2 to 0.25 ± 0.02 mm/year as the concentration of the inhibitor mixture in brine increases from 0 to 20 mg/L. This decrease is primarily associated with an increase in polarization resistance from 182 ± 5 to 1480 ± 50 Ohm cm^2^, which reduces anodic iron dissolution.

The change in ohmic resistance is small (Table A2). Table 1 summarizes the IE and IF values for Nalco EC 1010B under the conditions studied. The highest inhibition efficiency (IE) is achieved at a Nalco EC 1010B concentration of 20 mg/L in brine, corresponding to approximately 85% protection, depending on the method used to calculate the steel corrosion rate.

This concentration was selected for subsequent studies of the inhibitor’s effect on the steel corrosion rate in emulsified media. After 24 h in a corrosive solution containing 20 mg/L of the inhibitor, the corrosion rate decreases to 0.010 ± 0.001 mm/year, while IE increases to 99%. This effect is attributed to the formation of a continuous protective film on the steel surface, a well-established protective mechanism of organic inhibitors [1,10,23,31].

The diameters of the capacitive arcs (Figure 1b) and the impedance moduli (Figure 1c) increase markedly with inhibitor concentration and holding time, indicating improved corrosion resistance. As shown in Figure 1d, the phase-angle plots exhibit a plateau at approximately 50–60° (the phase angle of an ideal capacitive interface is −90°), suggesting only minor variation in capacitance among the samples examined.

**Figure 2:**
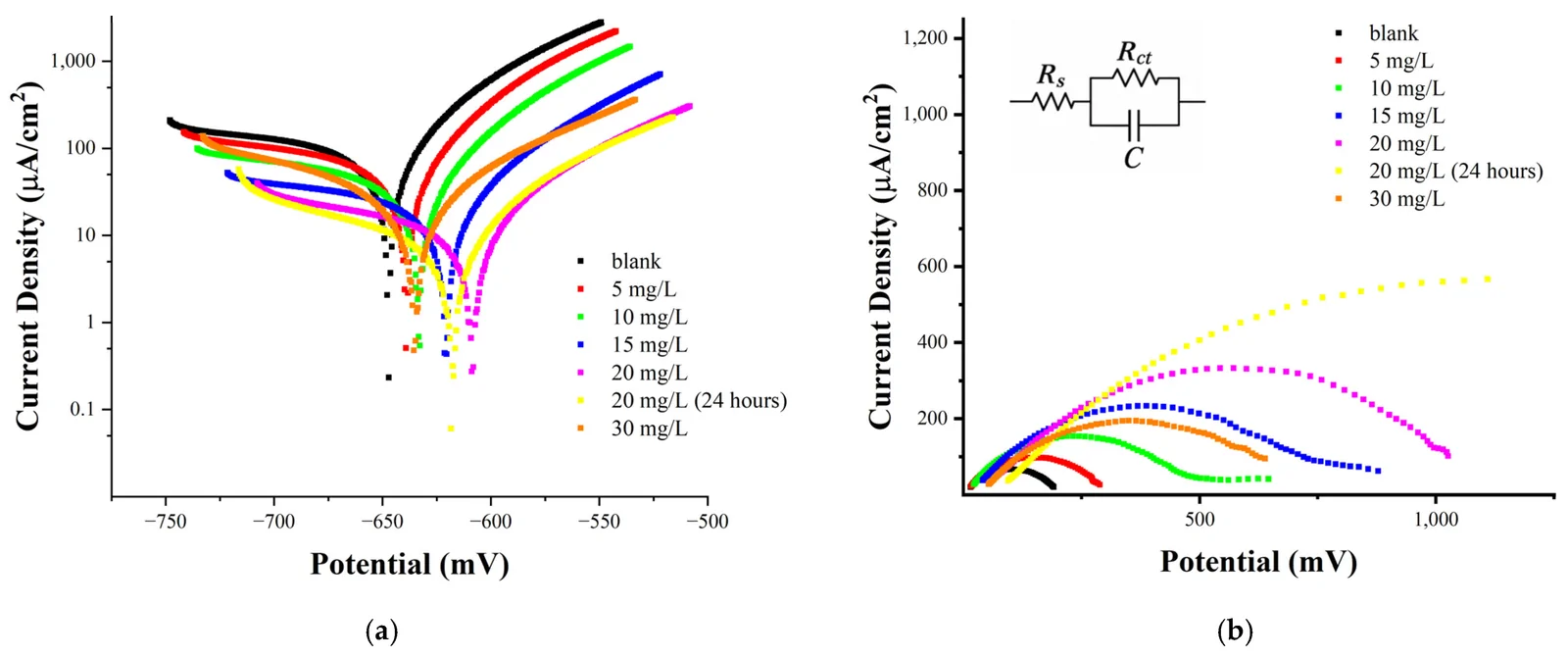

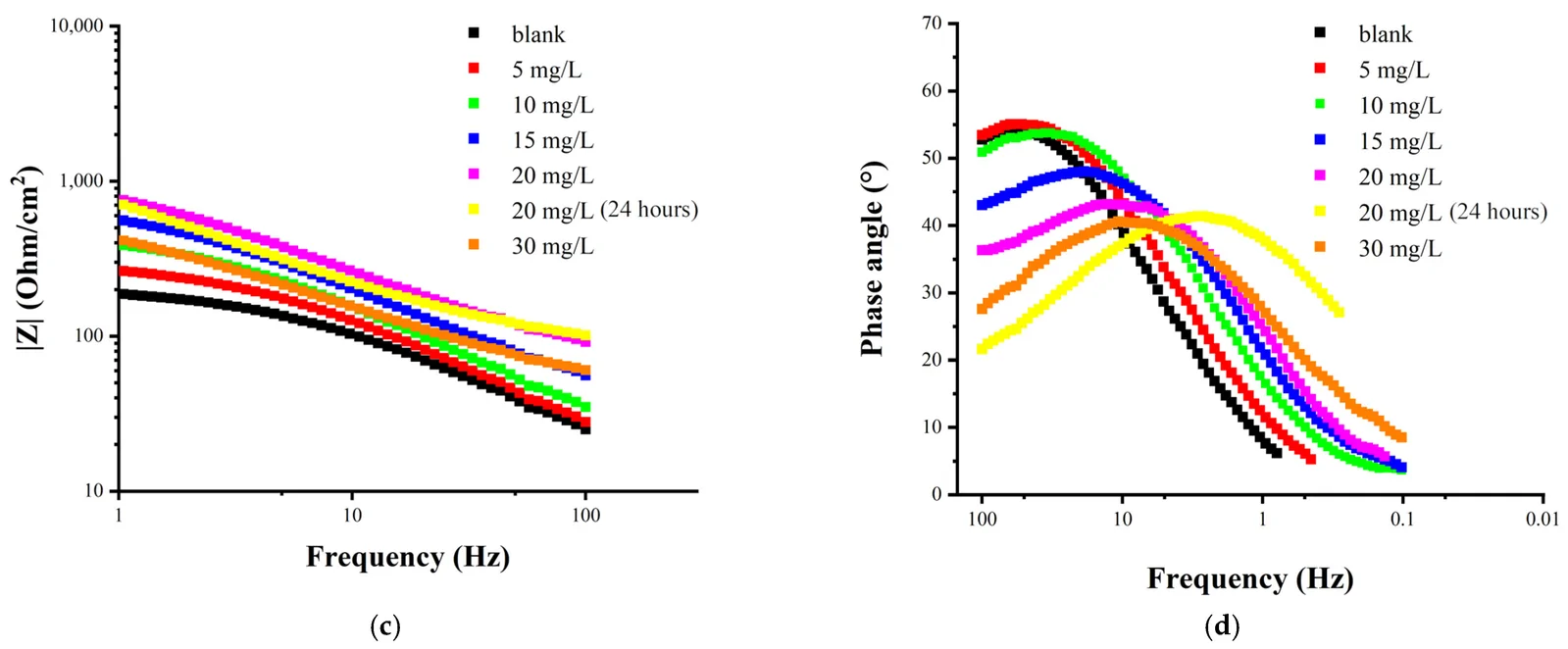
Tafel (**a**), Nyquist (**b**), Bode (**c**) and Phase angle (**d**) plots of steel coupons in brine in the presence of the Scimol OR 2001 inhibitor.

Figure 2 presents the Tafel (a), Nyquist (b), Bode (c), and phase-angle (d) plots for mild steel coupons in brine containing the Scimol OR 2001 inhibitor. The electrochemical measurements and corrosion rates calculated from the Tafel plots are presented in Table A3, while the EIS results are presented in Table A4.

The addition of the Scimol OR 2001 inhibitor mixture to brine reduces the steel corrosion rate. As the inhibitor concentration increases from 0 to 20 mg/L, the corrosion rate decreases gradually from 1.7 ± 0.2 to 0.23 ± 0.02 mm/year. This decrease is associated with an increase in polarization resistance from 182 ± 5 to 1500 ± 100 Ohm·cm^2^, reflecting inhibition of anodic iron dissolution. The change in ohmic resistance is negligible (Table A4). At an inhibitor concentration of 30 mg/L, the corrosion rate increases to 0.36 ± 0.03 mm/year. Table 2 presents the degree of protection and inhibition coefficient for the inhibitor mixture under the conditions studied. The highest degree of protection, approximately 85 ± 5%, is achieved at 20 mg/L.

After 24 h of exposure to a corrosive medium containing 20 mg/L of the Scimol OR 2001 inhibitor mixture, the steel corrosion rate decreased to 0.17 ± 0.02 mm/year, and the protection efficiency increased to 90 ± 5%. This effect is attributed to the formation of a robust protective film on the coupon surfaces [3]. The resulting protection is less effective than that achieved with the Nalco EC 1010B inhibitor mixture examined above.

### 2.2. The Impact of the Crude Oil Phase on the Corrosion Activity of the Electrolyte

Table 3 shows the polarization resistance (R) of steel coupons in crude oil–brine emulsified electrolytes of different compositions. In pure brine, the polarization resistance is 88 ± 6 Ohm cm^2^. In a stable micellar solution (MS), it increases approximately 15-fold to 1400 ± 25 Ohm cm^2^. Thus, the crude oil fractions in the stable emulsion act as corrosion inhibitors for steel [12,20,21]. In direct (O/B) and reverse (B/O) crude oil–brine emulsions, the effect of the crude oil fraction on polarization resistance is within the measurement error. The corresponding values are 95 ± 5 and 98 ± 8 Ohm cm^2^, respectively.

**Figure 3:**
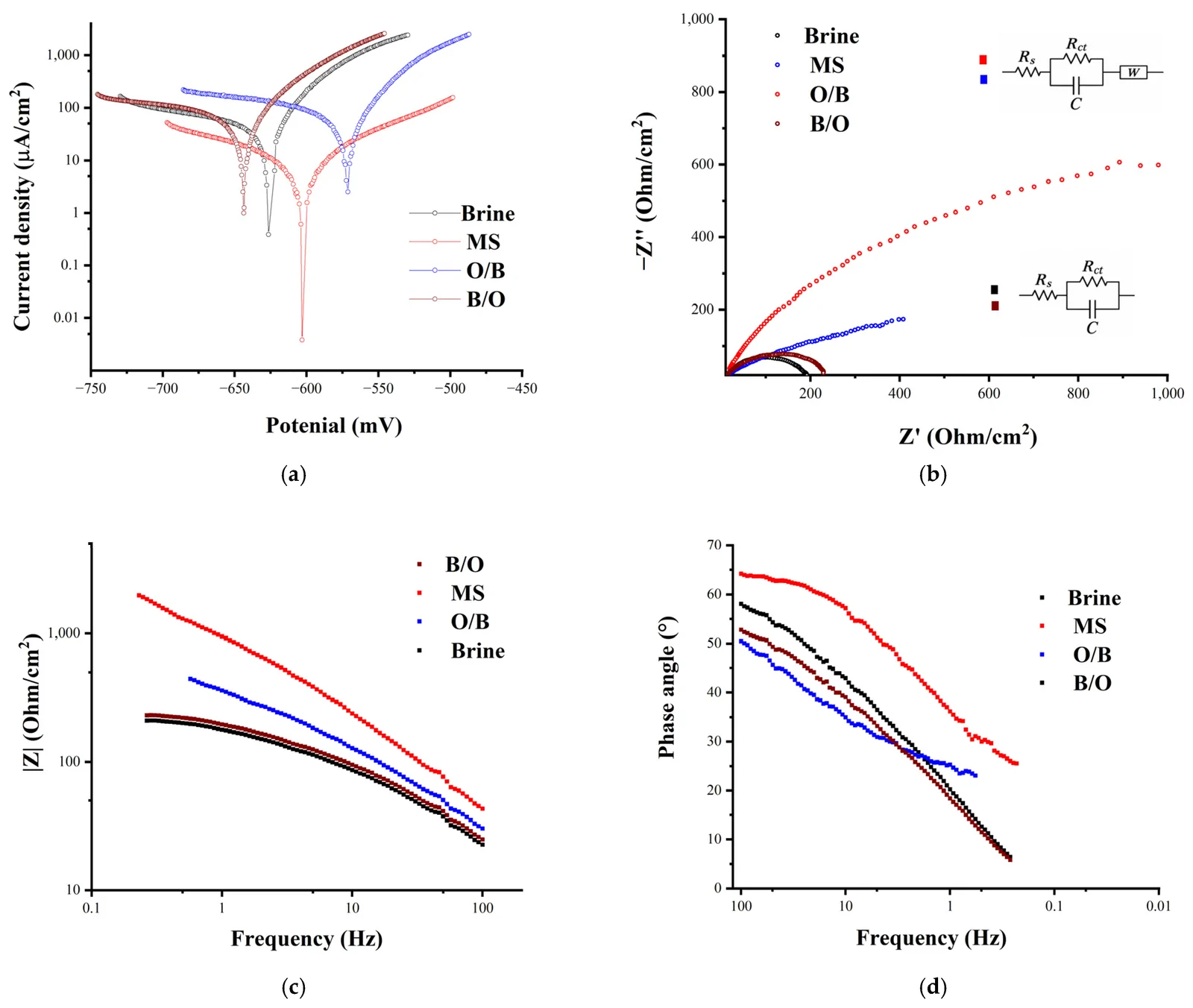
Tafel (**a**), Nyquist (**b**), Bode (**c**) and Phase angle (**d**) plots of steel coupons in various emulsified electrolytes.

Figure 3a presents the polarization curves recorded for steel coupons in emulsified electrolytes in Tafel coordinates. The Nyquist plots and equivalent circuits are shown in Figure 3b. The corrosion current density (i_COR_) and corrosion rate (CR) calculated from the Tafel plots are presented in Table A5. Table A6 presents the corrosion current density and corrosion rate obtained by EIS, together with the solution resistance (R_s_) and charge-transfer resistance (R_ct_).

A study of steel corrosion in crude oil–brine emulsions of varying composition showed that the corrosion rate in the stable emulsion decreased by an order of magnitude to 0.15 ± 0.03 mm/year relative to that in the initial brine (Table A1 and Table A2). In the presence of surfactants, the crude oil fractions in the stable emulsion act as organic corrosion inhibitors [6,9,10]. This decrease is accompanied by an anodic shift in potential and a doubling of the anodic Tafel slope (Table A5). These changes indicate slower anodic dissolution of iron to Fe^+^, which contributes to the lower corrosion rate.

The corrosion current density and corrosion rate of steel coupons in the direct emulsion and the brine fraction of the reverse emulsion are the same as those in pure brine. The electrochemical measurements therefore suggest that the crude oil fraction has no significant effect on steel corrosion under these conditions.

The results obtained by EIS are identical to those derived from the Tafel plots. The shape of the Nyquist plot for MS (red line in Figure 3b, open Warburg element) indicates the presence of an organic film on the metal surface. This film may prevent the diffusion of oxygen toward or away from the surface [11]. In this case, the organic film acts as a barrier that prevents the electrolyte from reaching the metal surface. This is reflected in an increase in charge-transfer resistance to 1900 ± 100 Ohm cm^2^. The Bode and phase-angle plots (Figure 3c,d) agree with the Nyquist plot analysis.

An open Warburg element is also visible in the Nyquist plot for the direct oil-in-water emulsion (blue line in Figure 3b). However, it does not correspond to an overall reduction in the steel corrosion rate. One possible explanation is that the oil film only partially covers the electrode surface because its position changes during the experiment [14]. Consequently, the measured corrosion rate remains the same as that in pure brine. The corrosion rate in the brine fraction of the reverse emulsion is likewise identical to that in pure brine.

The electrochemical methodology used to study steel corrosion in emulsified electrolytes depends on the composition and properties of the medium. The inhibitory effect of crude oil fractions on steel corrosion [23,25,31] was demonstrated only for the stable emulsion (MS). However, researchers frequently use multicomponent, stable emulsions to investigate the effect of an organic phase on the steel corrosion rate. Emulsion stability depends on the addition of a surfactant, which also acts as an inhibitor. This complicates the interpretation of data on the effects of organic inhibitors under these conditions and illustrates a limitation of studying steel corrosion inhibitors in stable emulsions.

In direct and reverse emulsions, the corrosion behavior of steel is governed by the composition and properties of the aqueous brine electrolyte. These emulsions are also the forms most likely to occur during oil refining and transportation [22].

Table 4 compares the results of this study with previously published data obtained under comparable experimental conditions [8,20]. The corrosion rates and corrosion currents are reproducible under these conditions. The present results corroborate previous findings and add to the existing evidence.

### 2.3. Corrosion Products Analysis and the Corrosion Process Mechanism

**Figure 4:**
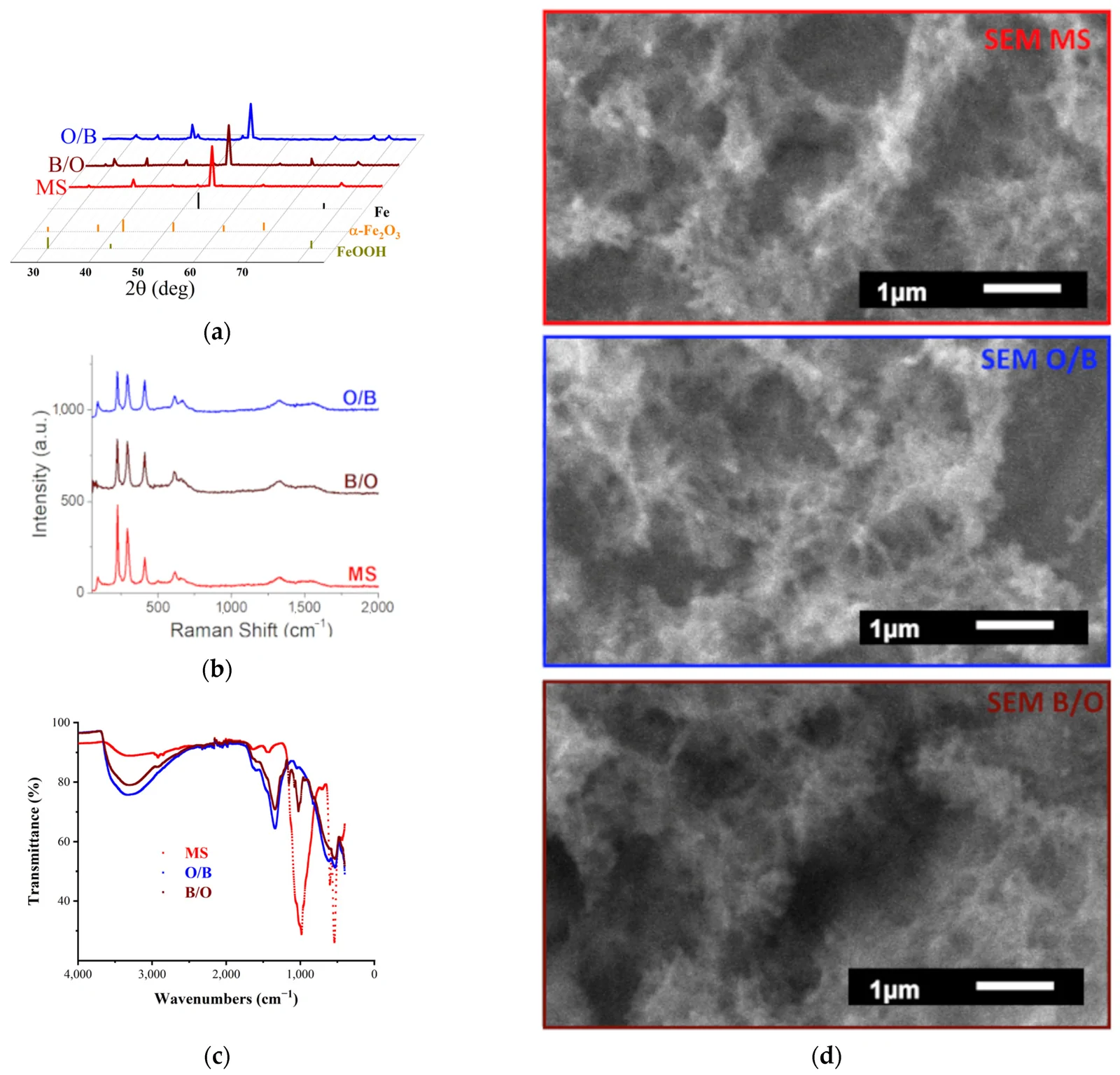
Corrosion products charaterization for steel coupons in different electrolytes: (**a**)—XRD, (**b**)—Raman spectra, (**c**)—FTIR spectra, and (**d**) SEM images.

The SEM images in Figure 4d show that the corrosion products on all coupons have an uneven, fibrous morphology with irregularly shaped inclusions. The diffraction peaks near 52° and 78° in the corresponding XRD patterns (Figure 4a) are assigned to the (110) and (200) planes of bcc α-Fe, respectively [32,33]. The peaks near 20°, 35°, 41°, 51°, and 69° correspond to the (111), (220), (311), (400), and (511) planes of Fe_2_O_3_·nH_2_O, respectively. The peaks near 30°, 43°, and 80° correspond to the (210), (301), and (711) planes of α-FeOOH, respectively. The XRD results confirm the presence of α-Fe_2_O_3_ and α-FeOOH deposits on the steel surface, which are the most stable phases in the near-neutral salt electrolyte [34]. The irregularly shaped inclusions indicate the presence of carbon compounds.

The composition of the corrosion products is further examined using the Raman spectra in Figure 4b. Regardless of electrolyte type, the α-Fe_2_O_3_ phase is indicated by three strong peaks at approximately 222, 281, and 399 cm^−1^ and two weaker bands at approximately 483 and 610 cm^−1^. The peaks at 281 and 399 cm^−1^ correspond to Fe–O vibrational modes involving two Fe atoms and O atoms. The positions and intensities of these bands are consistent with published data for α-Fe_2_O_3_ [35]. A C–C band at 1155 cm^−1^ indicates trace amounts of hydrocarbons from the crude oil fraction in the corrosion products [36].

The FTIR spectra of the steel coupons (Figure 4c) show characteristic peaks of Fe_2_O_3_, the primary corrosion product, in the 400–800 cm^−1^ range. Peaks near 1000, 1400, 1600, and 2900 cm^−1^ provide indirect evidence of an organic phase [18,19]. The marked differences in the spectrum obtained after testing in a stable emulsion may be attributed to the emulsion composition, including the presence of surfactants.

The multicomponent nature of the medium makes the data difficult to interpret. Nevertheless, the combined indirect evidence from several analytical techniques suggests the presence of an organic phase on the steel surface. Other explanations for the observations cannot be excluded.

The nature and composition of the corrosion products did not differ among the steel coupons studied. They were determined by the composition of the brine solution rather than the presence of the crude oil fraction. On the basis of these results, the corrosion mechanism was considered identical in all environments studied, regardless of petroleum phase content.

The data obtained support the same mechanism of steel corrosion [9,12] in all the emulsified electrolytes studied. Iron dissolution proceeds through a two-stage anodic process. First, water molecules adsorb onto the steel surface (1). They then dissociate into OH^−^ and H^+^ ions, while iron is oxidized to Fe^+^. Alongside the direct oxidation of iron, a reverse reaction reduces iron ions to atoms. In the second stage, Fe^+^ is oxidized to Fe^2+^ (2). The cathodic oxygen reduction reaction proceeds according to (3).

In solution, iron ions react with hydroxide ions to form a primary corrosion product (4). The unstable iron(II) hydroxide then reacts with dissolved oxygen in the electrolyte, initiating further corrosion and the formation of the expected final corrosion products (5–6). These products are deposited as residues.

(1) $${Fe}^{0}+H_{2}O_{ad}↔{Fe}^{+}{OH}^{-}+H^{+}+e^{-}$$

(2) $${Fe}^{+}\to {Fe}^{2+}+e^{-}$$

(3) $$O_{2}+2H_{2}O+{4e}^{-}\to {4OH}^{-}$$

(4) $${Fe}^{2+}+{2OH}^{-}\to {Fe(OH)}_{2}$$

(5) Fe(OH)2+12O2→2FeOOH↓+H2O

(6) $$2FeOOH\to {Fe}_{2}O_{3}↓+H_{2}O$$

### 2.4. Effect of the Inhibitors on Steel Corrosion in Emulsified Crude Oil–Brine Electrolytes

**Figure 5:**
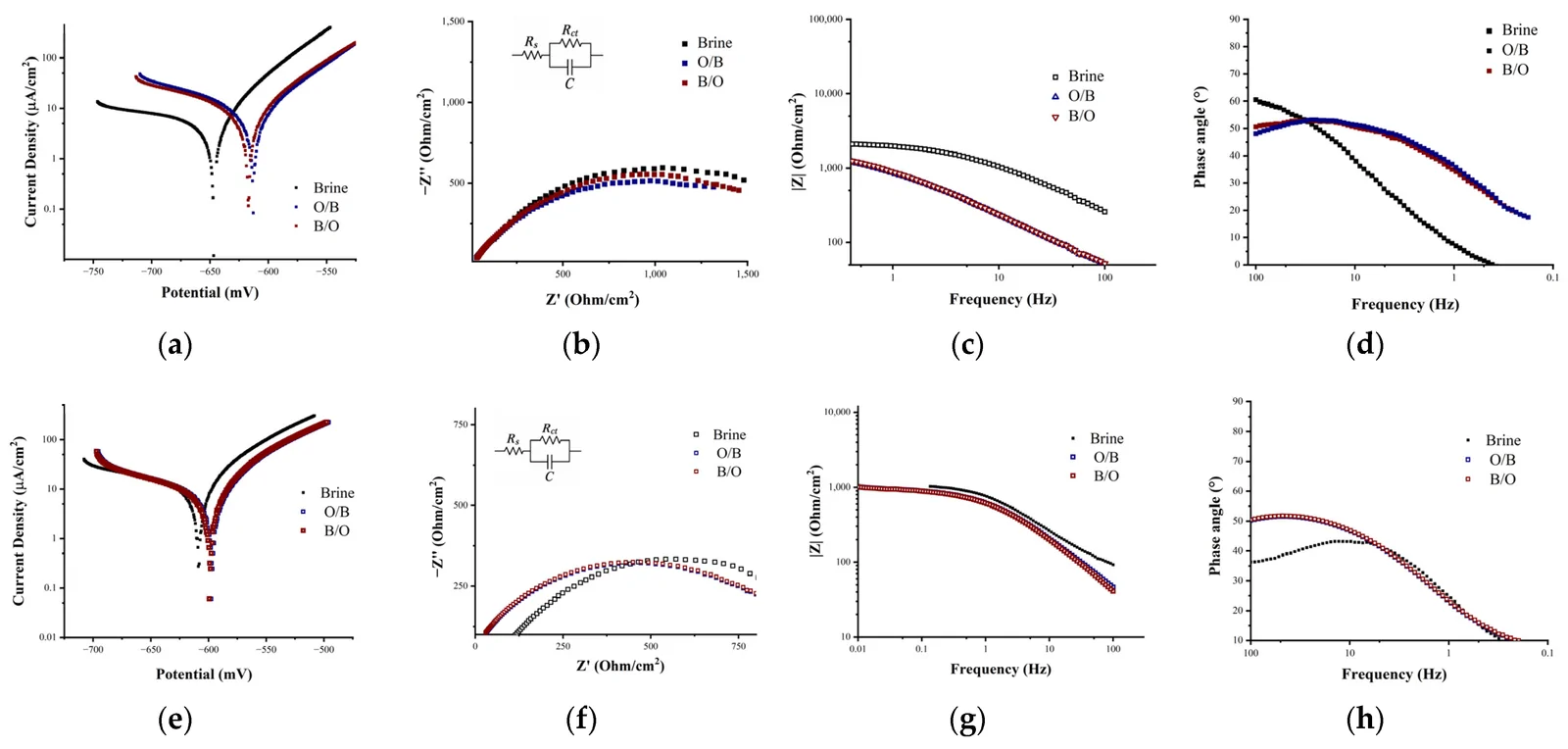
Tafel (**a**), Nyquist (**b**), Bode (**c**) and Phase angle (**d**) plots of steel coupons in emulsified electrolytes in the presence of 20 mg/L of the Nalco EC 1010B inhibitor; and Tafel (**e**), Nyquist (**f**), Bode (**g**) and Phase angle (**h**) plots of steel coupons in emulsified electrolytes in the presence of 20 mg/L of the Scimol OR 2001 inhibitor.

Figure 5 shows the Tafel and Nyquist plots for mild steel coupons in emulsified electrolytes in the presence of the Nalco EC 1010B and Scimol OR 2001 inhibitors. The electrochemical measurements and corrosion rates calculated from the Tafel plots are presented in Table A7, while the EIS results are presented in Table A8.

Regardless of the electrochemical method used, the corrosion rate of steel in all media examined remains at 0.20 ± 0.02 mm/year within experimental error. This value is the same in emulsified electrolytes, regardless of how the emulsion is prepared, and in brine tested under similar conditions. Thus, the crude oil fraction does not make a significant contribution to the inhibition efficiency of Nalco EC 1010B in emulsified media. The same pattern is observed for Scimol OR 2001, although its effect is somewhat less pronounced. Electrochemical data on the effects of organic inhibitors similar in composition to Nalco EC 1010B and Scimol OR 2001 on steel corrosion in brine can therefore be applied to both direct and reverse crude oil–brine emulsions.

The inhibitor mixture used in this study is polar and insoluble in crude oil. Its main components are amide derivatives of unsaturated C_18_-fatty acids and tetraethylenepentamine. These compounds exhibit pronounced surface activity because they contain both polar functional groups and long hydrocarbon chains. When the mixture contacts the steel surface, its nitrogen-containing and amide groups adsorb onto active iron sites, forming strong coordination bonds. The hydrophobic chains orient toward the liquid phase, forming a thin, impermeable film that acts as a barrier between the metal and the corrosive medium [37,38]. This layer is oleophilic and can promote the formation of an additional organic film from crude oil, providing further protection against penetration by the corrosive brine solution. The combined action of the inhibitor mixture and precipitation of the crude oil phase produces an organic film on the metal surface. This film acts as a barrier between the metal and the corrosive solution, reducing the rate of electrochemical metal dissolution. In the long term, a higher degree of protection is expected in an emulsified medium than in pure brine.

Therefore, the effect of organic corrosion inhibitors on mild steel corrosion in crude oil–brine emulsions can be assessed by studying their performance in an equivalent aqueous brine solution. This allows the inhibitor concentration required to reduce corrosion to the desired level to be determined. The crude oil fraction in the industrial medium is expected to provide further long-term protection.

## 3. Materials and Methods

### 3.1. Electrolytes

The media investigated included an aqueous brine solution and crude oil–brine emulsions prepared from it. The brine simulates formation water from the Kalamkas oil field (Mangystau region, Kazakhstan) and contains NaCl, NaBr, CaCl_2_, MgCl_2_, BaCl_2_, KBr, SrCl_2_, and KH_2_PO_4_ at concentrations of 67.8, 0.23, 36.7, 17.7, 0.23, 0.6, 1.15, and 1.7 g/L, respectively. All chemicals were supplied by RUSCHIM LLC (Moscow, Russia) and used without further purification. The brine pH is approximately 6. The high salt concentrations reflect the geographical and reservoir characteristics of the field and contribute to the high corrosivity of the brine. The crude oil phase consisted of light crude oil from the same field. Its density at 20 °C was 854.2 kg/m^3^ (33.2° API), its kinematic viscosity was 28.0 mm^2^/s, and its dynamic viscosity was 0.03 Pa·s.

The effect of the crude oil phase on inhibition efficiency was studied using the commercial corrosion inhibitors Nalco EC 1010B and Scimol OR 2001. The main active components of Nalco EC 1010B are unsaturated fatty acid amides and their reaction products with TetraEthylenePentAmine (TERA). These components contain amide and amine functional groups (–CONH–, –NH_2_, –NH–) and long C_18_ hydrocarbon chains. The primary inhibiting agent in Scimol OR 2001 is N-alkyl (tall oil fatty acid) polyethylenepolyamine. A mixture of aromatic hydrocarbons, including naphthalene (C_10_H_8_), promotes the formation of a hydrophobic film and passivation of the steel surface. A mixture of dimethylbenzene and ethylbenzene is used as a solvent because of the polarity of the inhibitor components. The selected inhibitor blends represent the most common class of industrial inhibitors used in primary oil processing units. These facilities experience severe corrosion because their emulsions contain substantial amounts of residual water and crude oil. Given the brine composition, the findings may be applicable to a wide range of organic corrosion inhibitors under conditions close to those encountered in industry. The use of industrial blends allows most components of the system to be considered while limiting variability.

**Figure 6:**
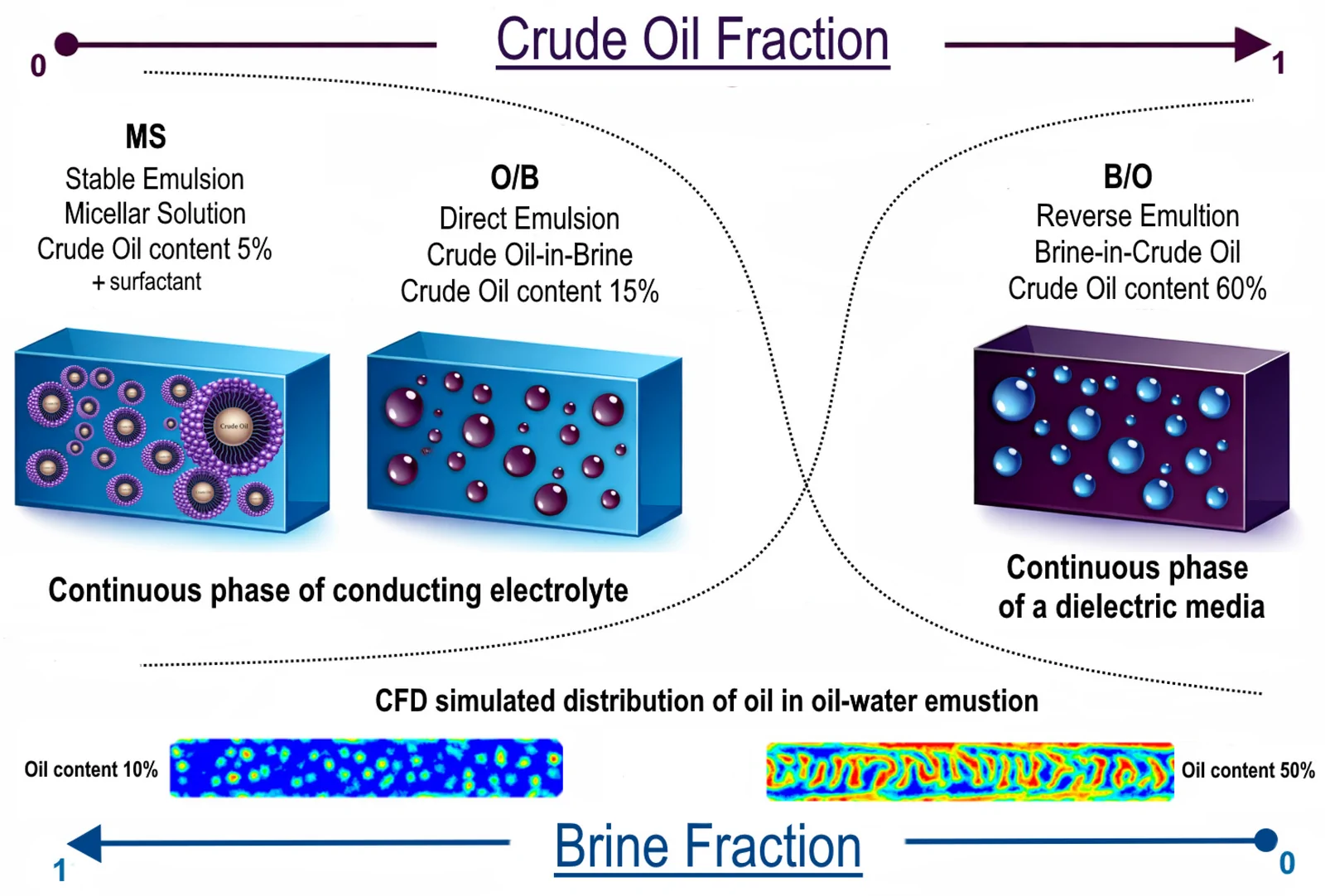
Schematic representation of the composition of emulsions depending on the phase ratio of the brine and crude oil fractions.

The use of electrochemical techniques [5,18,28,29] to determine steel corrosion rates in emulsions of crude oil fractions and brine is complicated by the heterogeneity and low electrical conductivity of the electrolyte, as oil behaves as a dielectric medium. Depending on the ratio of brine to petroleum fractions, three types of emulsions with different compositions and properties can be distinguished. These include two limiting cases [11,12,20], direct emulsions (oil-in-brine, O/B) and reverse emulsions (brine-in-oil, B/O), as illustrated in Figure 6, as well as stabilized emulsions prepared using emulsifying agents [8,12,21]. Direct and reverse emulsions are most commonly encountered in petroleum refining and transportation systems [22]. However, electrochemical studies predominantly use stable emulsions as electrolytes, typically prepared with chemical emulsifiers, including surfactants [8,12,24].

Electrochemical measurements were carried out using four electrolytes: brine, a stable brine–oil emulsion containing no more than 5 vol.% oil, a direct crude oil-in-brine emulsion containing no more than 15 vol.% oil, and the brine phase separated from a reverse brine-in-crude oil emulsion with an initial crude oil content of more than 60%. Table 5 lists the labels and compositions of the electrolytes studied.

### 3.2. Electrochemical Protocol

#### 3.2.1. Pretreatment

Emulsions with different compositions and properties require different approaches to electrochemical measurement. Measuring the electrochemical characteristics of steel corrosion requires sufficient electrolyte conductivity. Because petroleum fractions are predominantly dielectric, a continuous conductive phase may be absent from brine–crude oil emulsions. The aqueous brine serves as the electrolyte. Direct electrochemical measurements are therefore feasible only in direct (O/B) and stabilized (MS) emulsions with a continuous conductive phase [7].

**Figure 7:**
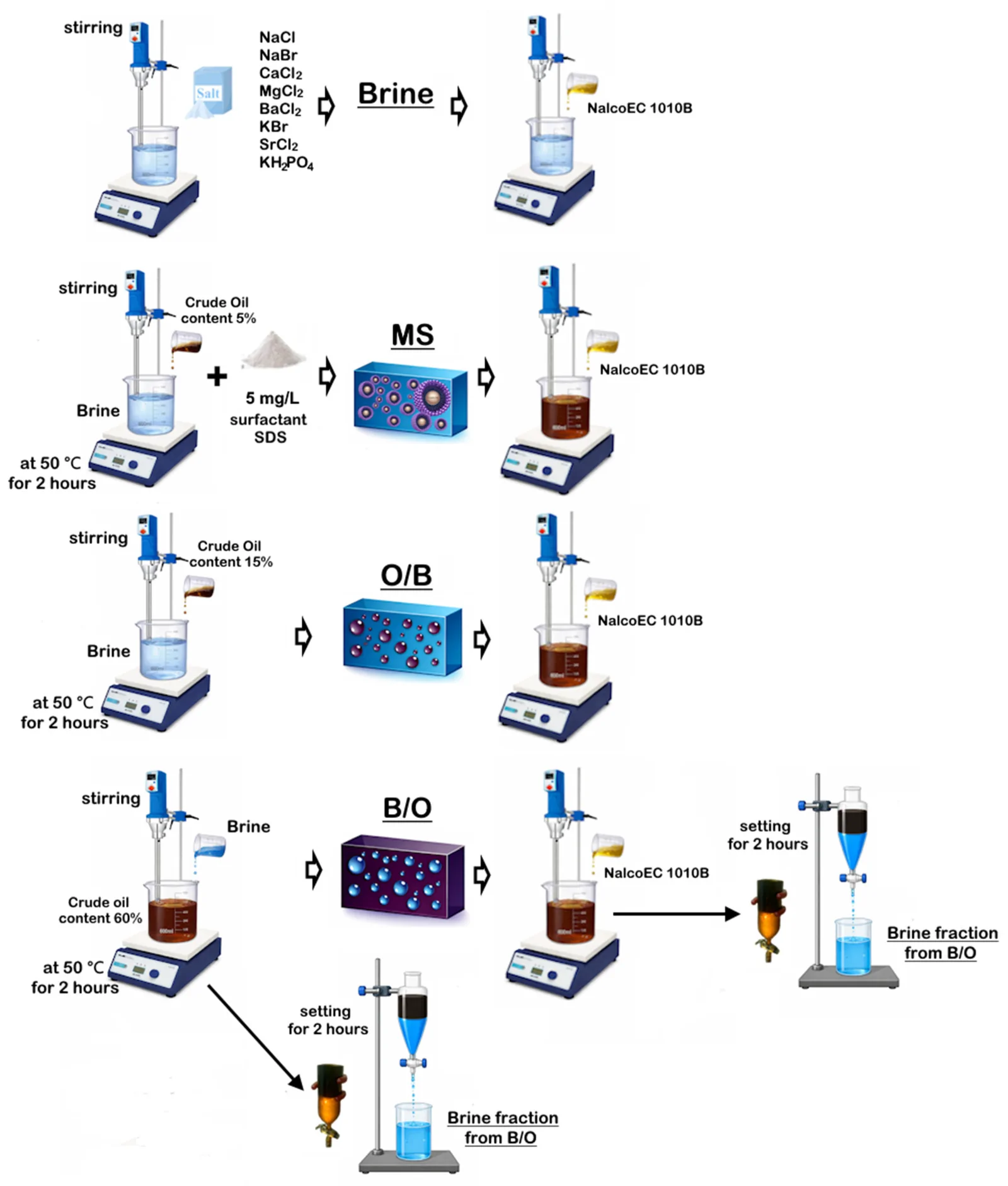
Electrolyte pretreatment diagram with approaches for its preparation.

The emulsions were prepared by stirring mixtures of brine and crude oil with short-term heating at 50 °C for 2 h (Figure 7). Sodium dodecyl sulfate was used as an emulsifying agent at a concentration of 5 mg/L to stabilize the emulsions and prepare the micellar solution (MS).

Electrochemical measurements in reverse emulsions (B/O) with a crude oil content exceeding 50% are not feasible because they lack a continuous conductive electrolyte phase [7]. Preliminary phase separation followed by electrochemical testing of the aqueous brine phase is therefore proposed (Figure 7). The brine was separated from the crude oil fraction using a separatory funnel after the emulsion had been allowed to stand for 2 h.

The effective concentration of the inhibitor mixture was determined in brine before the crude oil fraction was added. In studies evaluating the inhibitor’s effect on steel corrosion, the inhibitor was first dissolved in the emulsion under investigation. A schematic of the pretreatment protocol for the electrolyte component of the emulsified medium is shown in Figure 7.

#### 3.2.2. Electrochemical Test

The corrosion rate was determined using two independent electrochemical techniques: polarization curve measurements (Tafel plot analysis) and electrochemical impedance spectroscopy (EIS). To optimize experimental time and material use, the protocol involved stabilizing the open-circuit potential, recording anodic and cathodic polarization curves, and then acquiring impedance spectra. Each experiment was performed in triplicate for every set of parameters to ensure reproducibility.

**Figure 8:**
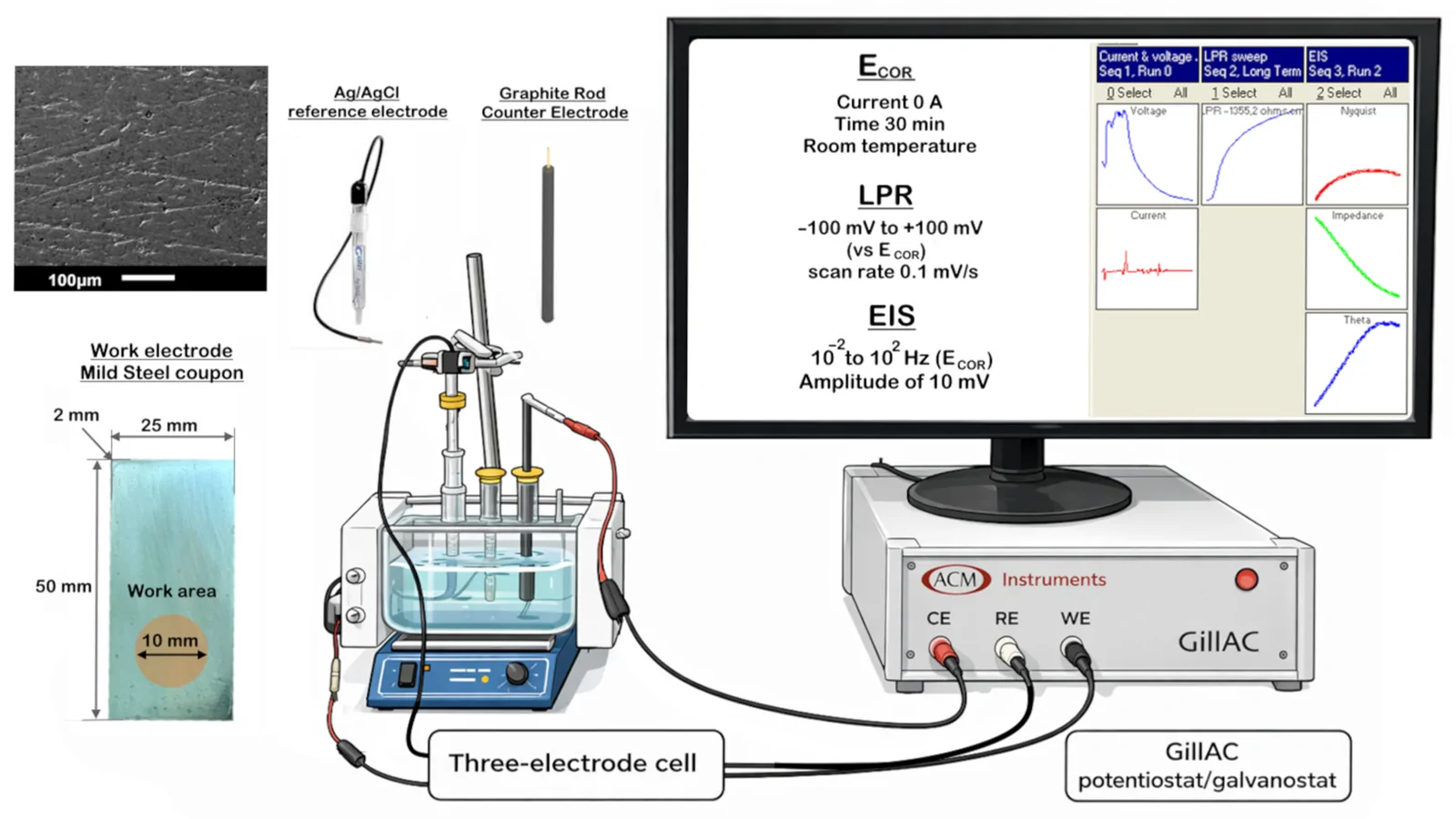
External appearance of the electrochemical cell and steel coupon.

Polarization curves and impedance spectra were recorded using a GillAC potentiostat/galvanostat (ACM Instruments, Grange-over-Sands, UK). All measurements were performed in a conventional 500 mL three-electrode glass cell. A saturated Ag/AgCl electrode served as the reference electrode, and a graphite rod served as the counter electrode. The working electrode was a mild steel coupon measuring 50 × 25 × 2 mm, with an exposed working area of 0.785 cm^2^. Figure 8 shows the electrochemical cell and steel coupons.

The working electrode was prepared in accordance with ASTM G1-03 [39]. The steel surface was first ground using 100 and 600 grit abrasive papers (Mirka LTD, Jeppo, Finland), then polished manually with 1500, 2000, and 2500 grit abrasives. The samples were rinsed with distilled water and isopropyl alcohol, dried, and conditioned at 23 ± 2 °C and 50 ± 5% relative humidity for at least 16 h. Before electrochemical measurements, the average surface roughness was measured using a profilometer. The mean roughness did not exceed 0.050 ± 0.005 μm, satisfying the requirements for EIS-based corrosion rate determination. The electrochemical cell was assembled immediately after surface preparation.

The open-circuit potential (E_COR_) was allowed to stabilize for 30 min in the electrolyte before measurements [5,18]. All measurements were performed at room temperature, approximately 25 °C. The electrochemical research protocol draws on published work on the corrosion behavior of steel in emulsified media [6,8,14,28]. The reliability of the data is supported by result reproducibility and the precision of values obtained through statistical analysis. The relatively high rates of metal corrosion affect the sample surface morphology during the test. This, in turn, negatively impacts the reproducibility and accuracy of the results obtained. Consequently, the data acquisition time is kept to a minimum, sufficient only for processing by GillAC software (Version 5). Polarization curves for Tafel analysis were recorded from −100 to +100 mV relative to E_COR_ at a scan rate of 0.1 mV/s. Linear regions were selected from −50 to −25 mV for the cathodic branch and from +25 to +50 mV for the anodic branch, both relative to E_COR_. The curves were used to determine the corrosion current density (i_COR_) and corrosion rate (CR) [28].

Nyquist, Bode, and phase-angle plots were obtained from EIS measurements at E_COR_ over a frequency range of 10^−2^ to 10^2^ Hz with a 10 mV amplitude [28,29,40]. The electrolyte composition and the research methods (rapid corrosion test) do not provide a protective film formation on the steel surface, which would require the extension of the recorded frequency region. The data were analyzed using an appropriate equivalent circuit model. Despite the limited frequency range, the data allowed comprehensive analysis and calculation of all resistances using the proposed equivalent circuits.

The inhibition efficiency (IE) and inhibition factor (IF) for steel corrosion were calculated using Equations (7) and (8), respectively [9].

(7) $$IE=\frac{{CR}_{without\;inh}-{CR}_{with\;inh}}{{CR}_{without\;inh}}\times 100\%$$

(8) $$IF=\frac{{CR}_{without\;inh}}{{CR}_{with\;inh}}$$

where ${CR}_{without\;inh}$ is the steel corrosion rate in a corrosion solution without inhibitor, and ${CR}_{with\;inh}$ is the steel corrosion rate in a corrosion solution with inhibitor (other conditions are similar for both cases).

### 3.3. Corrosion Products Analysis

The corrosion morphology of the steel was examined using a JEOL JSM 6610 LV (JEOL Ltd., Akishima, Japan) scanning electron microscope equipped with an INCA Energy 450 microanalysis system (Oxford Instruments, High Wycombe, UK).

X-ray diffraction analysis was performed using a DRON-4 (Bourevestnik, Saint Petersburg, Russia) automated diffractometer with a cobalt anode. The operating conditions were 35 kV and 20 mA in θ–2θ mode, with a detector speed of 2°/min. The diffraction patterns were interpreted using the ICDD powder diffraction card database.

Raman spectra were collected using a Solver Spectrum Raman spectrometer (NT-MDT Spectrum Instruments, Zelenograd, Russia) with an excitation wavelength of 635 nm. Spectra were acquired for 100 s from 2 μm regions over the range of 0–2000 cm^−1^, with a resolution of ±4 cm^−1^.

## 4. Conclusions

This study evaluated the effects of organic inhibitors on mild steel corrosion in brine and crude oil–brine emulsions using Tafel polarization and electrochemical impedance spectroscopy. The measurement approach depended on the emulsion type. Direct and stable emulsions with a continuous conductive phase could be tested directly, whereas reverse emulsions required separation of the brine phase before electrochemical measurement due to their low conductivity.

In brine, both inhibitors achieved a protection efficiency above 80% at a concentration of 20 mg/L. At this concentration, corrosion rates in the inhibitor-containing direct emulsion and the brine phase separated from the reverse emulsion were approximately 0.20–0.25 mm/year. These values were comparable to those obtained in inhibited brine under similar conditions, indicating that the crude oil fraction had no significant effect on the measured inhibitor performance in these media.

Stable emulsions presented an additional limitation because the surfactant used for stabilization also affected steel corrosion. Its contribution was difficult to distinguish from that of the inhibitor mixture. Determining the effective inhibitor concentration in brine therefore provides a practical basis for evaluating protection in direct emulsions and in the aqueous phase of reverse emulsions under the conditions studied.

The effective concentration of 20 mg/L in the aqueous brine phase may also be relevant when phase separation occurs during crude oil transportation. During prolonged exposure, an oil-derived surface film may provide additional protection.

## Figures and Tables

**Table 1:** The IE and the IF for the Nalco EC 1010B under the conditions studied depending on its content in brine.

Nalco EC 1010B Content, mg/L	5	10	15	20	20 (24 h)
IF	1.2 ± 0.1	1.4 ± 0.1	4.0 ± 0.1	7.2 ± 0.1	180 ± 10
IE, %	15 ± 5	30 ± 5	75 ± 5	85 ± 5	99 ± 5

**Table 2:** The IE and the IF for the Scimol OR 2001 under the conditions studied, depending on its content in brine.

Scimol OR 2001 Content, mg/L	5	10	15	20	20 (24 h)	30
IF	1.3 ± 0.1	1.8 ± 0.1	4.3 ± 0.1	7.4 ± 0.1	10.0 ± 0.5	4.3 ± 0.1
IE, %	25 ± 5	45 ± 5	75 ± 5	85 ± 5	90 ± 5	75 ± 5

**Table 3:** Polarization resistance values for steel coupons in crude oil–brine electrolytes.

Electrolyte	Label	Polarization Resistance R, Ohm cm^2^	E_COR_, mV
Brine	B	88 ± 6	−620 ± 10
Direct Emulsion	O/B	95 ± 5	−560 ± 10
Reverse Emulsion Brine Phase	B/O	98 ± 8	−640 ± 10
Micellar Solution	MS	1400 ± 20	−600 ± 10

**Table 4:** Comparison of corrosion rate values for a steel coupon in emulsified electrolytes.

No	Conditions	CR, mm/Year	i_COR_, µA/cm^2^	Ref.
1	Carbon Steel. NaHCO_3_ (0.02 M) and NaCl (0.02 M) were mixed using 0.2 wt% dioctyl sulfosuccinate sodium salt to form the anionic surfactant agent. The pH value of solutions was 8.57.	-	142–181	[8]
2	Carbon Steel. NaHCO_3_ (0.02 M) and NaCl (0.02 M) were mixed using 0.2 wt% dioctyl sulfosuccinate sodium salt to form the anionic surfactant agent. The oil–water emulsion consisted of 10% engine oil (1 g/L), Tween 80 (0.5 g/L), Span 80 (0.5 g/L) and distilled water. The pH value of solutions with 10% oily phase was 8.62.	-	155–245	[8]
3	Brine	1.52 ± 0.25	140 ± 8	Present study
4	Brine—Crude Oil emulsions	1.47 ± 0.25	135 ± 10	Present study
5	Mild Steel. The composition of the brine solution was (0.1 mol NaCl + 0.01 mol NaHCO_3_+ 0.01 mol CaCl_2_)/L to mimic the produced water in the CO_2_-EOR field condition. Unstable mixture with 60% oil phase.	1.5	-	[20]
6	Mild Steel. The composition of the brine solution was (0.1 mol NaCl + 0.01 mol NaHCO_3_+ 0.01 mol CaCl_2_)/L to mimic the produced water in the CO_2_-EOR field condition. Stable mixture with 10% oil phase.	0.1	-	[20]
7	Stable emulsion (MS)	0.15 ± 0.02	15 ± 2	Present study

**Table 5:** Label and composition of electrolyte samples investigated.

Electrolyte Samples	Label	Composition	
		Brine, %	Crude Oil, %
Brine	B	100	-
Direct emulsion crude oil-in-brine	O/B	˃75	˂15
Stable Emulsion Micellar solution	MS	˃95 +5 mg/L CH_3_(CH_2_)_11_OSO_3_Na Sodium Dodecyl Sulfate (SDS)	˂5
Reverse Emulsion Brine-in-crude oil	B/O	˂40 (pure brine in electrochemical test)	˃60

## Data Availability

Data will be made available upon request.

## Abbreviations

The following abbreviations are used in this manuscript:

EIS	electrochemical impedance spectroscopy
MS	Micellar Solution
B/O	Brine in Crude Oil Emulsion
O/B	Crude Oil in Brine Emulsion

## Appendix A

Appendix A contains the electrochemical data used to evaluate mild steel corrosion in brine and crude oil–brine emulsions. Table A1, Table A2, Table A3 and Table A4 report the Tafel and EIS results for brine tested with Nalco EC 1010B and Scimol OR 2001. Table A5 and Table A6 provide the results for emulsions without inhibitors, while Table A7 and Table A8 provide the results for emulsions containing the inhibitors.

**Table A1:** Corrosion current density and corrosion rate values for a steel coupon in brine in the presence of the Nalco EC 1010B inhibitor according to the Tafel Plots calculation method.

Nalco EC 1010B Content, mg/L	Coupon	E_COR_, mV	Anodic Tafel Slope, mV	Cathodic Tafel Slope, mV	i_COR_, µA/cm^2^	CR, mm/Year
blank	Mild Steel	−650 ± 10	40 ± 5	115 ± 5	158 ± 25	1.8 ± 0.2
5		−640 ± 10	40 ± 5	90 ± 5	134 ± 15	1.5 ± 0.2
10		−640 ± 10	40 ± 5	115 ± 5	112 ± 8	1.3 ± 0.1
15		−640 ± 10	40 ± 5	115 ± 5	39 ± 2	0.45 ± 0.02
20		−640 ± 10	40 ± 5	90 ± 5	21 ± 1	0.25 ± 0.02
20 (24 h)		−610 ± 10	40 ± 5	115 ± 5	0.8 ± 0.1	0.010 ± 0.001

**Table A2:** Corrosion current density and corrosion rate values for a steel coupon in brine in the presence of the Nalco EC 1010B inhibitor according to the EIS calculation method.

Nalco EC 1010B Content, mg/L	Coupon	i_COR_, µA/cm^2^	CR, mm/Year	R_s_, Ohm cm^2^	R_ct_, Ohm cm^2^	Double-Layer Area Capacity, F·10^4^
blank	Mild Steel	142 ± 20	1.7 ± 0.2	6 ± 5	182 ± 5	2.1 ± 0.1
5		127 ± 15	1.5 ± 0.2	6 ± 5	205 ± 5	3.1 ± 0.3
10		93 ± 10	1.10 ± 0.15	5 ± 5	282 ± 5	5.4 ± 0.3
15		24 ± 4	0.32 ± 0.03	5 ± 5	460 ± 5	1.0 ± 0.1
20		18 ± 4	0.20 ± 0.02	10 ± 5	1480 ± 50	0.35 ± 0.03
20 (24 h)		0.7 ± 0.1	0.008 ± 0.001	160 ± 10	22,900 ± 500	(4.6 ± 0.2) × 10^−2^

**Table A3:** Corrosion current density and corrosion rate values for a steel coupon in brine in the presence of the Scimol OR 2001 inhibitor according to the Tafel Plots calculation method.

Scimol OR 2001 Content, mg/L	Coupon	E_COR_, mV	Anodic Tafel Slope, mV	Cathodic Tafel Slope, mV	i_COR_, µA/cm^2^	CR, mm/Year
blank	Mild Steel	−650 ± 10	40 ± 5	115 ± 5	158 ± 25	1.8 ± 0.2
5		−640 ± 10	40 ± 5	120 ± 5	123 ± 15	1.4 ± 0.2
10		−630 ± 10	45 ± 5	100 ± 5	80 ± 5	0.93 ± 0.07
15		−620 ± 10	45 ± 5	120 ± 5	45 ± 3	0.45 ± 0.03
20		−600 ± 10	40 ± 5	90 ± 5	21.2 ± 0.7	0.25 ± 0.02
20 (24 h)		−610 ± 10	40 ± 5	90 ± 5	16.0 ± 0.7	0.18 ± 0.02
30		−630 ± 10	70 ± 5	110 ± 5	33.5 ± 1.7	0.40 ± 0.03

**Table A4:** Corrosion current density and corrosion rate values for a steel coupon in brine in the presence of the Scimol OR 2001 inhibitor according to the EIS calculation method.

Scimol OR 2001 Content, mg/L	Coupon	i_COR_, µA/cm^2^	CR, mm/Year	R_s_, Ohm cm^2^	R_ct_, Ohm cm^2^	Double-Layer Area Capacity, F·10^4^
blank	Mild Steel	142 ± 20	1.7 ± 0.2	6 ± 5	182 ± 5	2.1 ± 0.1
5		110 ± 15	1.3 ± 0.2	10 ± 5	260 ± 5	2.2 ± 0.3
10		75 ± 5	0.95 ± 0.15	5 ± 5	550 ± 10	3.4 ± 0.3
15		33.5 ± 2.5	0.40 ± 0.05	10 ± 5	1000 ± 50	1.8 ± 0.3
20		19.2 ± 0.7	0.23 ± 0.02	10 ± 5	1500 ± 100	3.1 ± 0.2
20 (24 h)		14.7 ± 0.7	0.17 ± 0.02	10 ± 5	1820 ± 100	3.4 ± 0.2
30		36.3 ± 2.5	0.36 ± 0.03	20 ± 5	720 ± 50	2.3 ± 0.2

**Table A5:** Corrosion current density and corrosion rate values for a steel coupon in emulsified electrolytes according to the Tafel Plots calculation method.

Electrolyte	Coupon	E_COR_, mV	Anodic Tafel Slope, mV	Cathodic Tafel Slope, mV	i_COR_, µA/cm^2^	CR, mm/Year
B	Mild Steel	−620 ± 10	50 ± 5	145 ± 10	135 ± 6	1.52 ± 0.21
O/B		−560 ± 10	35 ± 5	115 ± 10	130 ± 5	1.53 ± 0.21
MS		−600 ± 10	80 ± 5	120 ± 10	15 ± 2	0.15 ± 0.02
B/O		−640 ± 10	35 ± 5	120 ± 10	127 ± 5	1.47 ± 0.21

**Table A6:** Corrosion current density and corrosion rate values for a steel coupon in emulsified electrolytes according to the EIS calculation method.

Electrolyte	Coupon	i_COR_, µA/cm^2^	CR, mm/Year	R_s_, Ohm cm^2^	R_ct_, Ohm cm^2^	Double-Layer Area Capacity, F·10^4^
B	Mild Steel	140 ± 8	1.52 ± 0.25	7 ± 5	360 ± 50	2.4 ± 0.3
O/B		135 ± 10	1.47 ± 0.25	5 ± 5	250 ± 50	4.5 ± 0.5
MS		14 ± 2	0.15 ± 0.03	7 ± 5	1900 ± 100	2.6 ± 0.3
B/O		135 ± 10	1.46 ± 0.25	5 ± 5	250 ± 50	2.5 ± 0.3

**Table A7:** Corrosion current density and corrosion rate values for a steel coupon in emulsified electrolytes in the presence of inhibitors according to the Tafel Plots calculation method.

Electrolyte	Inhibitor	E_COR_, mV	Anodic Tafel Slope, mV	Cathodic Tafel Slope, mV	i_COR_, µA/cm^2^	CR, mm/Year
B	Nalco EC 1010B	−640 ± 10	40 ± 5	90 ± 5	21.2 ± 2.7	0.22 ± 0.02
O/B		−620 ± 10	40 ± 5	95 ± 5	18.2 ± 1.7	0.20 ± 0.02
B/O		−620 ± 10	40 ± 5	90 ± 5	19.5 ± 1.8	0.21 ± 0.02
B	Scimol OR 2001	−600 ± 10	40 ± 5	90 ± 5	21.2 ± 0.7	0.25 ± 0.02
O/B		−600 ± 10	40 ± 5	90 ± 5	20.8 ± 1.5	0.22 ± 0.02
B/O		−600 ± 10	40 ± 5	90 ± 5	20.7 ± 1.5	0.22 ± 0.02

**Table A8:** Corrosion current density and corrosion rate values for a steel coupon in emulsified electrolytes in the presence of inhibitors according to the EIS calculation method.

Electrolyte	Inhibitor	i_COR_, µA/cm^2^	CR, mm/Year	R_s_, Ohm cm^2^	R_ct_, Ohm cm^2^	Double-Layer Area Capacity, F·10^4^
B	Nalco EC 1010B	18 ± 4	0.20 ± 0.02	10 ± 5	1480 ± 50	0.35 ± 0.03
O/B		17 ± 4	0.20 ± 0.02	10 ± 5	1450 ± 50	0.37 ± 0.03
B/O		19 ± 4	0.20 ± 0.02	10 ± 5	1460 ± 50	0.39 ± 0.03
B	Scimol OR 2001	19 ± 2	0.23 ± 0.02	10 ± 5	1500 ± 100	0.31 ± 0.02
O/B		18 ± 2	0.24 ± 0.03	10 ± 5	1450 ± 100	0.37 ± 0.02
B/O		18 ± 2	0.24 ± 0.03	10 ± 5	1450 ± 100	0.35 ± 0.02
